# Leaving Before Completion of Treatment Among Alcohol-Intoxicated, College-Aged Emergency Department Patients

**DOI:** 10.7759/cureus.38265

**Published:** 2023-04-28

**Authors:** David P Betten, Kirk J Trentham, Bill Corser, Kristen N Owen

**Affiliations:** 1 Department of Emergency Medicine, Sparrow Health System, Lansing, USA; 2 Department of Emergency Medicine, McLaren Greater Lansing, Lansing, USA; 3 Department of Medicine, Michigan State University College of Osteopathic Medicine, East Lansing, USA

**Keywords:** medical decision making capacity, against medical advice, alcohol use disorder, binge drinking, alcohol intoxication, college-aged alcohol consumption, emergency department elopement

## Abstract

Background

Heavy alcohol use among college-aged students is common and may lead to Emergency Department (ED) visits. A review of alcohol-intoxicated presentations to a single ED was performed to characterize these encounters and identify factors associated with leaving before treatment completion.

Methodology

Electronic medical records were reviewed for patients aged 18 to 25 years over a nine-month study period who presented to a university-affiliated ED with isolated alcohol intoxication and were subsequently discharged or left before completion of treatment. The frequency and characteristics of these individuals were compared using chi-square analysis. A series of controlled logistic and multinomial regression models were conducted to examine the predictive significance of potentially confounding variables (age, gender, time and day of presentation, method of hospital arrival, and triage level) associated with premature ED departure and length of stay. Measured ethanol levels and vital sign abnormalities at the time of leaving the ED were identified.

Results

Four hundred sixty-four patients aged 18 to 25 years presented with isolated alcohol intoxication over the study period. A higher frequency of leaving without completion of treatment was noted in college-aged alcohol-intoxicated individuals compared to the general adult ED population (17.9% versus 3.5%; *P *< 0.01). Abnormal vital signs (10.5%) and elevated ethanol levels before ED departure when measured (85.2%) were not uncommon. Variables significantly associated with leaving before completion of treatment included arrival by means other than emergency medical service (EMS)/police, lower triage levels, and 11 p.m. to 7 a.m. hospital departure.

Conclusions

Based on these results, intoxicated college-aged individuals are at high risk for leaving EDs before care completion. The development of targeted protocols to minimize this occurrence and utilizing these ED encounters to consider addressing unhealthy drinking behaviors may be helpful.

## Introduction

Heavy and excessive alcohol consumption and the resultant negative health effects are an undeniable presence in our society. Rates of potentially dangerous alcohol usage are particularly high among full-time college students. In 2019, in the United States, 33% of students aged 18 to 22 years met the criteria for binge drinking behavior in the preceding month (five or six drinks in one sitting for women or men, respectively) with 9.7% of male college students and 6.8% of female students considered heavy alcohol users (binge drinking >5 times per month) [1]. The risks associated with excessive alcohol consumption among college-aged students are numerous, including unintentional injuries, sexual and nonsexual assaults, poor academic performance, financial and legal difficulties, and an increased prevalence of anxiety and depression [2]. Despite these risks, college students frequently may be unaware of or minimize these concerns and accept heavy alcohol use as part of the college experience and necessary to fit into the social construct [3,4].

Given the frequency of irresponsible drinking behavior noted in this relatively alcohol-naïve population, Emergency Department (ED) visits are not uncommon. Physicians and nurses tasked with managing a busy ED may experience frustration and lack of satisfaction in caring for these individuals who are simply *drunk* and need to *sleep it off* [5]. As a result of this, intoxicated patients are often considered to be of a lower acuity and, therefore, may perceive to be of little benefit in both prolonged observations and educating these individuals on the inherent acute dangers or long-term risks associated with these behaviors [6]. Given the presumed benign nature of these patient encounters, the lack of necessary medical interventions, and providers' frequent ambivalence toward the education of young, intoxicated individuals, patients leaving before assessment and care completion are not uncommon. Patients who are significantly intoxicated and who leave the ED without adequate levels of physician assessment are of concern from a patient's safety standpoint, given the lack of clear judgment they possess and often unclear understanding of reasons for being initially transported to the ED.

To understand the scope of this issue with greater clarity, we performed a retrospective study to characterize college-aged (18-25 years), alcohol-intoxicated ED encounters in a university-affiliated, community-based setting and examined predictive influences of care delivery characteristics among patients leaving before treatment completion.

## Materials and methods

Study design, setting, and participants

Following institutional review board (IRB) approval, a retrospective review of electronic medical records was performed over nine months (August 2018 to April 2019) at a community-based ED (98,000 visits annually) located close to a large university made up of approximately 40,000 students. Medical records were queried for all patients between 18 and 25 years of age at the time of ED presentation who had a primary complaint recorded by triage nursing staff of *alcohol intoxication* or *acute intoxication*. For comparative purposes, an additional search was performed to quantify the frequency of leaving before treatment completion among the adult population who did not have alcohol intoxication reported to triage nursing staff as the reason for their ED presentation. 

To evaluate the patients presenting with solely primary alcohol intoxication in our 18- to 25-year age study group, patients with traumatic injury, concomitant substance use other than marijuana, suicidal ideation/attempt, and sexual assault were excluded. Patients with nausea, vomiting, and altered mental status were included because these symptoms were consistent with alcohol intoxication. Individuals admitted to the hospital were also excluded from the study. A search of the study subject’s medical charts was performed to determine if repeat visits to the same ED occurred within 72 hours following their initial alcohol intoxication-related visit.

Data collection

For 18- to 25-year-old patients presenting with intoxication, information was recorded regarding age, gender, time of arrival, length of stay, disposition, method of arrival, and triage level assigned. For individuals who left before treatment completion, the last vital signs obtained before departure from the ED (pulse, respiratory rate, blood pressure, and pulse oximetry), and measured ethanol level if performed in the ED or immediately before arrival by EMS or police, were recorded.

Patient disposition was classified as being either *discharged* or *left before treatment completion.* Patients considered to have left before treatment completion included patients who had ultimate disposition classified in the electronic chart as *left without completion of service*, *left without being seen*, and those marked as *eloped*. No patients in our study group were reported to have *left against medical advice.* Method of patient arrival was classified based upon nursing triage notes as EMS, police, private vehicle, taxi, or other/unknown. Triage level was recorded based on the Emergency Medical Severity Index using a 1 through 5 scale, with the lowest acuity being level 5. Ethanol levels were included if measured in the ED as a breathalyzer or serum specimen or if reported as being taken via breathalyzer by police or EMS before arrival, with the time of recording as when reported in the electronic medical record.

For individuals who left before treatment completion and had an ethanol level measured, an estimated ethanol level at the time of the patient departing the ED was extrapolated using a presumed ethanol elimination rate of 20 mg/dL/hour subtracted from their initial ethanol level [7]. For this study, the last vital signs recorded before discharge were considered abnormal by study investigators if the pulse rate was <50 or >110 beats per minute, respirations were <10 or >20 breaths per minute, mean arterial pressure was <65 mmHg, or pulse oximetry of < 94% was recorded.

Data analysis

Before data analyses, G*Power 3.1.6 software was used to generate initial pre-analytic minimal sample size calculations for the hypothesized *main effect* influence of gender affiliation on variations in ED elopement occurrences [8]. These calculations indicated that a minimal total sample size of at least 84 discrete adults (i.e., 42 males and 42 females) would afford the study team an acceptable level of statistical power (80%) to detect statistically significant proportionate variations between the two gender sample subgroups, as reported by Dugas et al. [9].

To evaluate sample subgroup and outcome distributional patterns, a series of descriptive statistics, cross-tabulation charts, and graphical examinations of data were generated. For most analyses, continuous variables (e.g., age category and ED length-of-stay outcome) were conservatively categorized into equivalent-sized tertile sample subgroups. A series of simple chi-square tests of independence and Pearson *r* bivariate correlations were generated to evaluate variations of factors stratified by outcomes [10].

Logistic regression was performed to examine the relationship between predictors with log odds of leaving ED before completion of treatment. Multinomial regression procedures were performed to assess the relationship between predictors associated with categorical ED length-of-stay occurrences [11]. For all analytic procedures, a two-tailed *P*-value of less than 0.05 indicated statistical significance. Descriptive and inferential analytic procedures were performed using SPSS Statistics for Windows, Version 28.0 (IBM Corp., Armonk, NY, USA) [12].

## Results

Over the nine-month study period, 11,752 individual encounters occurred among 18 to 25-year-olds, of which 464 had an ED presentation due to isolated alcohol intoxication (3.9%) (Table 1). The mean age of patients leaving before completion of treatment was 20.3 years with 56.3% occurring among men. Of the 464 encounters, there were 43 involving individuals with more than one visit (range 2-5 visits). The frequency in which our alcohol-intoxicated young adult study group left before treatment completion was 17.9% (83/464). This is significantly greater than the 3.5% (2,213/62,566) rate of all adult patients aged 18 years or older who did not present with alcohol intoxication (*P *< 0.01) and left before treatment completion. There were no return visits to the ED within three days for patients who left before treatment completion.

**Table 1:** Alcohol intoxicated patients (18-25-year-olds) demographic, presentation, and disposition characteristics. EMS, emergency medical service

Encounter characteristics	Number of patients, *n* (%)
Disposition	
Discharge	381 (82.1)
Left before treatment completion	83 (17.9)
Repeat patient visits	
No	421 (90.7)
Yes	43 (9.3)
Age (years)	
18 or 19	215 (46.3)
20 to 23	193 (41.6)
24 to 25	56 (12.1)
Gender	
Male	261 (56.3)
Female	203 (43.8)
Arrival mode	
EMS or police	378 (81.5)
Private vehicle	59 (12.7)
Other/None listed	27 (5.8)
Triage level	
Level 1 or 2	182 (39.2)
Level 3	193 (41.6)
Level 4 or 5	85 (18.3)
Time of presentation	
2300 to 0659 hours	336 (72.4)
0700 to 1459 hours	42 (9.1)
1500 to 2259 hours	86 (18.5)
Day of week presenting	
Monday through Thursday	98 (21.1)
Friday through Sunday	366 (78.9)
Time of discharge or left before treatment completion	
0700 through 1459 hours	160 (34.5)
1500 through 2259 hours	68 (14.7)
2300 through 0659 hours	236 (50.9)

Alcohol-intoxicated 18- to 25-year-olds presented more frequently between the 11 p.m. and 7 a.m. (72.4%), with most of these visits occurring between Friday and Sunday (78.9%). With regard to the time of presentation, alcohol intoxication was the sole complaint in 12.5% (350/2,788) of the young adult study population who presented between 11 p.m. and 7 a.m. in comparison to only 1.3% (114/8,964) during the 7 a.m. to 11 p.m. (*P *< 0.01). Between 11 p.m. and 7 a.m., 18.9% (66/350) of young adults with alcohol intoxication left before treatment completion compared to 5.9% (143/2,438) non-alcohol-intoxicated young adults (*P *< 0.01). Rates of those who left before treatment completion did not vary significantly among those with alcohol intoxication as a chief complaint who presented during 11 p.m. to 7 a.m. compared to other times (16.7%, 19/114; *P *= 0.67).

A significantly greater number of intoxicated young adult patients who left before treatment completion arrived in the ED in a manner other than by EMS or police were assigned to a lower triage level (3, 4, or 5) or were patients who left the ED between 11 p.m. and 7 a.m. (Table 2). Gender, age, and day or time of presentation were not identified as predictors of leaving with treatment completion. Regarding the overall length of stay of all college-aged primary alcohol-intoxicated patients, a significantly longer length of stay, regardless of disposition, was identified in patients presenting on weekends, a higher triage level acuity, as well as among those who were discharged during 7 a.m. to 3 p.m. (Table 3). When controlling for confounders using multivariate regression modeling for disposition and multinomial regression modeling as factors impacting length of stay, similar demonstrated variables were found to be significant (Tables 4-5).

**Table 2:** Chi-square cross-tabulation tests for significance for variables related to ED encounters involving patients leaving before treatment completion. ED, emergency department; df, degrees of freedom; EMS, emergency medical service

	Patient discharged	Patient left before treatment completion, *n* (%)	Chi-square	df	Significance
Arrival means			15.618	2	<0.001
EMS/police	337 (83.2)	41 (69.5)			
Private vehicle	51 (12.6)	8 (13.6)			
Other/unknown	17 (4.2)	10 (16.9)			
Triage level			14.979	2	<0.001
1 or 2	171 (42.4)	11 (19.3)			
3	166 (41.2)	27 (47.4)			
4 or 5	66 (16.4)	19 (33.3)			
Time of leaving the ED			20.720	2	0.002
0700-1459 hours	153 (37.8)	7 (11.9)			
1500-2259 hours	62 (15.3)	6 (10.2)			
2300-0659 hours	190 (46.9)	46 (78.0)			
Age (years)			0.177	2	0.915
18 or 19	189 (46.7)	26 (44.1)			
20 to 23	167 (41.2)	26 (44.1)			
24 or 25	49 (12.1)	7 (11.9)			
Gender			0.259	1	0.611
Female	179 (44.2)	24 (40.7)			
Male	226 (55.8)	35 (59.3)			
Time of ED presentation			2.682	2	0.262
0700-1459 hours	40 (9.9)	2 (3.4)			
1500-2259 hours	75 (18.5)	11 (18.6)			
2300-0659 hours	290 (71.6)	46 (78)			
Weekend or weekday presentation			2.401	1	0.121
Monday-Thursday	81 (20.0)	17 (28.8)			
Friday-Sunday	324 (80.0)	42 (71.2)			

**Table 3:** Chi-square cross-tabulation tests for significance for variable related to ED length of stay. ED, emergency department; df, degrees of freedom; EMS, emergency medical service

	0- to 183-minute length of stay, *n* (%)	184- to 329-minute length of stay, *n* (%)	333-minute length of stay or greater, *n* (%)	Chi-square	df	Significance
Arrival means				4.535	4	0.338
EMS/Police	121 (78.6)	123(79.4)	134 (86.5)			
Private vehicle	21 (13.6)	23 (14.8)	15 (9.7)			
Other/Unknown	12 (7.8)	9 (5.8)	6 (3.9)			
Triage level				23.504	4	<0.001
1 or 2	43 (28.1)	63 (41.2)	76 (49.4)			
3	66 (43.1)	70 (45.8)	57 (37)			
4 or 5	44 (28.8)	20 (13.1)	21 (13.6)			
Time of leaving the ED (hours)				131.379	4	<0.001
0700-1459	15 (9.7)	38 (24.5)	107 (69)			
1500-2259	30 (19.5)	24 (15.5)	14 (9)			
2300-0659	109 (70.8)	93 (60.0)	34 (21.9)			
Age (years)				9.229	4	0.056
18 or 19	71 (46.1)	82 (52.9)	62 (40)			
20 to 23	70 (45.5)	56 (36.1)	67 (43.2)			
24 or 25	13 (8.4)	17 (11.0)	26 (16.8)			
Gender				2.407	2	0.300
Female	71 (46.1)	72 (46.5)	60 (38.7)			
Male	83 (53.9)	83 (53.5)	95 (61.3)			
Time of ED presentation (hours)				2.915	4	0.572
0700-1459	16 (10.4)	16 (10.3)	10 (6.5)			
1500-2259	32 (20.8)	26 (16.8)	28 (18.1)			
2300-0659	106 (68.8)	113 (72.9)	117 (75.5)			
Weekend or weekday presentation				8.675	2	0.013
Monday-Thursday	31 (20.1)	23 (14.8)	44 (71.6)			
Friday-Sunday	123 (79.9)	132 (85.2)	11 (28.4)			

**Table 4:** Binary multivariate logistic regression results for dichotomous outcome disposition category - left before treatment completion or discharge.

Variable	Wald chi-square	df	Significance
Arrival mode	13.320	2	.001
Triage level	13.913	2	< .001
Time period at discharge	19.600	2	< .001
Age category	1.049	2	0.592
Gender	2.051	1	0.152
Time of presentation	1.717	2	0.424
Weekday or weekend presentation	2.791	1	0.095

**Table 5:** Multivariate multinomial logistic regression modeling results for categorical length of stay.

Variable	Wald chi-square	df	Significance
Triage level	28.645	4	0.001
Weekend or weekday presentation	9.755	2	0.13
Time period of discharge or elopement	143.385	4	<0.001

Of those patients who had vital signs recorded during their ED stay and left before completion of treatment, 10.5% (8/76) had a pulse greater than 110 beats per minute reported as their last recorded vital signs. No other vital sign abnormalities were noted. Of the 27 individuals in which an ethanol level was ascertained, an extrapolated level of greater than 80 mg/dL was noted in 23 individuals at the time of leaving without completion of treatment was recorded, with 13 of these individuals having levels above 200 mg/dL. 

## Discussion

High-risk alcohol consumption is estimated to contribute to 140,000 deaths annually in the United States, with problematic drinking likely to become even more prevalent, given a recent rise in heavy alcohol usage related to the COVID pandemic [13,14]. Although alcohol remains one of the greatest preventable risk factors for death among adults aged under 35 years, societal perception of heavy alcohol consumption as a relatively benign phenomenon and behavior often embraced as one begins their transition into adulthood would suggest healthcare professionals will continue to routinely encounter alcohol-related ED visits well into the foreseeable future [15]. This unhealthy pattern of binge alcohol consumption has been shown in this single-center ED patient population to result in heavy ED utilization among intoxicated young people, especially during the night and on weekends. These visits are associated with a rate of elopement before complete evaluation at a rate over five times that of the general adult ED population. 

The utilization of EDs among binge-drinking college students has been reported across college campuses throughout the United States and abroad. Turner and Shu described 16% of ED visits by students at a midsize university to be alcohol-related, representing 0.7% of the student body over a single year [16]. Similarly, a campus-based ambulance service in a large northeastern university found that 17% of transports among college-age students were found to be related to alcohol overuse [17]. Given the high frequency of alcohol use among college students, intoxicated patients are often dismissed as being of low acuity. The potential for underestimating the likelihood of serious disease occurring among those with apparent simple alcohol intoxication may exist with it being not uncommon for physicians to obtain imaging studies and implement other therapeutic interventions based upon their clinical assessment [18]. At times, the presence of alcohol intoxication itself may be incorrectly assumed. In a large review of nearly 30,000 patients, a diagnosis of alcohol intoxication was found to be inaccurate in 6.4% of presumed ethanol intoxication in patients presenting with mental status changes with 1 in 10 of these visits requiring hospital admission [19].

In our study, the percentage of patients leaving before completion of ED treatment (17.9%) was high but not inconsistent with that seen in other hospital settings. In a Swiss study, up to 49% of alcohol-intoxicated patients left without being seen with male gender, age under 26, nighttime presentation, and low triage levels as primary risk factors [9]. In the United States, a university-affiliated ED reported a left without the treatment rate of 15% for those with alcohol intoxication (compared to 2.9% of all patients) with a 50% reduction in this rate following a triage-initiated, protocol-based approach involving nurses, physicians, and advanced practice providers established to reduce the number of unsafe premature patient departures [20].

An ED visit for isolated heavy alcohol consumption may be a window of opportunity to educate receptive individuals on the risk of binge drinking behaviors. Encounters with healthcare providers among healthy college students are infrequent, and this may be a crucial first step to inducing long-term changes in individuals with unhealthy drinking behaviors. An understanding that high-risk alcohol consumption in college-aged individuals increases the risk of heavy alcohol usage and alcohol dependence later in life supports early interventions among those who may not consider themselves to be at high risk [21]. Simple interventions at the time of ED presentation for alcohol intoxication utilizing brief motivational techniques with post-ED discharge follow-up have shown promising results in producing a sustained reduction in abnormal drinking behaviors [22,23].

Barriers to providing education and intervention in the ED may exist, unfortunately, as the high rate of individuals presenting during overnight hours in our study often coincides with a period when ED case managers and social workers may be unavailable or staffed at lower levels. This may lead to challenges in finding adequate time to provide in-depth counseling and resources to these patients. Efforts to align appropriate staffing resources with periods when higher volumes of alcohol-related presentations occur may offer a greater opportunity to benefit a large number of at-risk patients.

The frequency of patients leaving the ED prematurely with ethanol levels that were greater than >200 mg/dL, as well as the number of patients leaving with either the absence of vital signs recorded, or abnormalities of vital signs present, is concerning. Although the clinical effects of alcohol at levels such as these are individualized, assuming a lack of significant tolerance, many would likely lack the ability to make clear and nonhindered assessments of their condition. While the risk of respiratory and cardiovascular compromise would be low in a patient ambulating independently from the ED, behavior patterns that alerted EMS providers or police to the necessity of medical care initially may be presumed to remain. Additionally, leaving the ED during nighttime hours, in an unfamiliar environment, may place these intoxicated individuals in an unsafe and potentially vulnerable state.

Limitations

Study limitations include a lack of certainty with regard to precise times in which patients left without completion of care hindering conclusions on length of stay and calculated ethanol levels at the time of leaving the ED before completion of treatment. It is uncertain if patients leaving without completion of care were left alone and independently or were accompanied by responsible and nonintoxicated friends or family, potentially leading to a less-risky discharge scenario. Rates of ethanol elimination do vary considerably and our elimination rate of 20 mg/dL/hour was chosen as a somewhat above-accepted average ethanol elimination rate among nondaily heavy drinkers to avoid overstating the frequency of individuals possessing elevated levels typically associated with impaired judgment at their time of elopement [7]. Additionally, serum ethanol levels were reported rather than whole blood ethanol measurements, which may lead to a potential overestimation of ethanol levels in patients who left the ED prematurely [24]. The exact times of ethanol levels obtained and inherent limitations of breathalyzers may have also contributed to the estimated rather than precise ethanol level we surmised to be present at the time of ED departure. While study investigators have anecdotally noted that the majority of ED visits related to ethanol consumption in this age group are university students, no attempts were made to verify the enrollment of study patients at the local university. No repeat visits following premature ED departure were noted among our patients; however, the possibility that a return visit occurred to an alternative medical center cannot be ruled out.

## Conclusions

ED visits by college-aged students for primarily isolated alcohol intoxication are not an uncommon event with the majority of visits found, not surprisingly, to occur more frequently on weekend nights extending into the early hours of the following morning. For receptive individuals, opportunities for patient education may be warranted with the recognition that persistent problematic drinking behaviors among college-aged students may have potentially both short- and long-term sequelae. Our study identified a rate of patients leaving the ED before care completion among those aged 18 to 25 years who present with alcohol intoxication significantly greater than the general ED population. Predictors of these higher rates of elopement included those arriving by non-EMS or police means, leaving between 11 p.m. and 7 a.m., and those triaged at lower levels. A heightened awareness of factors placing patients at risk of leaving before treatment completion and strategies to minimize this occurrence is important. Consideration of standardized pathways to provide adequate and more complete medical assessment and to ensure safe disposition plans are put into place for this potentially vulnerable patient population should be considered. 
